# The role of patient-reported outcome and experience measures in cardio-thoracic surgery

**DOI:** 10.1093/icvts/ivae025

**Published:** 2024-03-15

**Authors:** Cecilia Pompili, Bart Scheenstra, Carmelina Zirafa, Franca Melfi, Sabina De Rosis, Milena Vainieri, Kelvin Lau, Peyman Sardari Nia

**Affiliations:** Barts Thorax Centre, St Bartholomew Hospital, London, UK; Department of Cardiothoracic Surgery, Heart and Vascular Center, Maastricht University Medical Center, Maastricht, Netherlands; Minimally Invasive and Robotic Thoracic Surgery, Department of Surgical, Medical and Molecular Pathology and Critical Care Medicine, University of Pisa, Pisa, Italy; Minimally Invasive and Robotic Thoracic Surgery, Department of Surgical, Medical and Molecular Pathology and Critical Care Medicine, University of Pisa, Pisa, Italy; Management and Healthcare Laboratory, Institute of Management and Department EMbeDS, Scuola Superiore Sant’Anna, Pisa, Italy; Management and Healthcare Laboratory, Institute of Management and Department EMbeDS, Scuola Superiore Sant’Anna, Pisa, Italy; Barts Thorax Centre, St Bartholomew Hospital, London, UK; Department of Cardiothoracic Surgery, Heart and Vascular Center, Maastricht University Medical Center, Maastricht, Netherlands

**Keywords:** Quality of life, PROMs, PREMs, Patient satisfaction, Quality of care

## INTRODUCTION

Technological advancement in cardio-thoracic surgery has transformed the available treatments landscape, with improved survival rates but with the potential of substantial effect on the health-related quality of life (HRQoL). To promote patient-centred care, it is of paramount importance, as highlighted by several initiatives including Enhanced Recovery After Surgery (ERAS) Programmes [1], to incorporate patient-reported outcome measures (PROMs) into research and clinical care, in addition to traditional clinical outcomes [2].

Personalized care is now the core concept in many healthcare plans, including the British National Health System (NHS) one, focusing on giving people choice and control over the way their care is planned and delivered. It is based on ‘what matters’ to them and their individual strengths and needs. The availability of PROMs data helps clinicians to communicate the benefits and the risks of a treatment, giving the patient a clearer overview of potential alternatives tailored for his/her individual, which has been demonstrated to be rarely discussed in lung cancer care [3].

The concept of personalized care is directly linked to value-based healthcare, which aims to maximize the value of care provided for patients within available resources. The value-based healthcare is clearly based on patient-related outcomes rather than services volume or results and thereby the instrumental role of PROMs and patient-reported experience measures (PREMs) to quantify them.

## PATIENT-CENTRED CARE AND PATIENT-REPORTED EXPERIENCE MEASURES

Increasingly used during the recent decades, PROMs assess patient symptoms and HRQoL from the patient’s perspective to identify any treatment-related changes. Nevertheless, PREMs are psychometrically validated questionnaires used to capture patients’ perceptions of multiple aspects of their in-hospital or outpatient episodes of care. PREMs are designed to determine whether patients have experienced certain care processes rather than their satisfaction with the care received. PREMs provide insight into the quality of care during all the process of care delivered. They are effective ways of gathering patient feedback and can facilitate effectiveness and cost-effectiveness analysis to improve decision-making and service improvement. PREMs can also be used to improve services by identifying areas with substandard practice to drive service improvement and most importantly to build a patient-centred care reflecting the patient perspective and promoting direct patient engagement in care. There is also increasing evidence in demonstrating the positive correlations between patient experience with clinical effectiveness and patient safety, as well as adherence to recommended medication, treatments and use of screening services [4].

## USE OF PATIENT-REPORTED EXPERIENCE MEASURES IN CARDIO-THORACIC PRACTICE: ROUTINE CLINICAL CARE AND RESEARCH

In our speciality, patient-reported measures are still not routinely embedded into clinical practice [5], but initial evidences have demonstrated the potential roles in research and clinical care settings [6, 7].

As the cardio-thoracic surgery population is getting older and more comorbid over the last decades there is an increased risk of adverse postoperative events such as mortality and morbidity. To quantify and decrease these events, there is a growing need to identify, measure and predict relevant outcomes after elective surgery. To assist this quantification, the International Consortium for Health Outcomes Measurement (ICHOM) provides core outcome sets for different diseases including coronary artery disease and valvular heart disease [2]. A systematic review evaluating the use of PROs during recovery after adult cardiac surgery showed [8] similar challenges that are faced by cancer researchers [9], especially in implementing them in the clinical pathways.

Nevertheless, looking at the research setting, we witnessed increased efforts in determining the value of treatment for patients as demonstrated by the increased use of PROMs as primary end-points in clinical trials as recently published results by the VIOLET [6] or Adaura [10] trials. Several initiatives around the globe have been introduced to collect PROMs and PREMs data in nationally managed registries. In 2009, the UK Department of Health launched a mandatory initiative to measure and improve clinical quality by collecting and reporting PROMs from 4 key surgical interventions. In the Swedish healthcare system, the national quality registers are obliged to incorporate PROs for certification [11]. In the USA, the Hospital Consumer Assessment of Healthcare Providers and Systems (HCAHPS) is the nationally mandated and publicly available survey of inpatient experience and they are used primarily for national, regional and local comparisons between providers [12].

Black *et al.* [13] have shown that there is a positive correlation between experience and outcomes and that patients can distinguish between clinical effectiveness, safety and their experiences.

### How to use patient-reported measures for preoperative risk assessment and shared decision-making lesson learned from patient-reported outcome measures

Besides mortality and morbidity, postoperative HRQoL is deemed an important variable in core outcome sets by both patients and healthcare professionals. However, current clinical prediction models for cardiac surgery, using traditional risk factors, do not reliable predict postoperative HRQoL [14]. These models rely almost solely on physician-reported healthcare information as predictors (e.g. age, sex, surgery characteristics, comorbidity, biomarkers and imaging parameters) while lacking PROMs [15] such as HRQoL [16].

A systematic review that focused on predictors for postoperative HRQoL identified several modifiable predictors (e.g. alcohol use, body mass index/weight, depression, preoperative quality of life and smoking) that could be identified and targeted preoperatively to improve postoperative HRQoL [14]. Unfortunately, patient-reported health information (e.g. physical activity, weight loss, depression) and PROMs are rarely recorded in daily care [14]. As the consulting time is scarce, it is attractive to record PROMs prior to the consultation [17]. The use of this unique patient-reported health information is of great value for risk assessment and shared decision-making (SDM) [18].

SDM stimulates the exchange of knowledge between caregiver and patient and thereby equalizes the traditionally patriarchic relation. By taking patient preferences in account, SDM is considered an important step to select the right treatment for individual patients [19, 20]. As the incidence of postoperative events in cardiac surgery is high and patients become older and more comorbid, SDM is increasingly relevant during the preoperative period. Next, we will show how we implemented patient-reported health information and PROs in our preoperative work-up at the Department of Cardiothoracic Surgery from the Maastricht University Medical Center, in the Netherlands.

Patients who are referred to our department for a preoperative assessment are scheduled at our outpatient clinic for a preoperative assessment including the cardiothoracic surgeon, anaesthesiologist, ward physician, nurse and physiotherapist. One week before the assessment, our case manager calls patients to inform them about the appointment and to provide a personal login for a digital patient journey app (including information about preoperative optimization, different surgical procedures, risk of complications and postoperative rehabilitation). In addition, patients receive an electronic questionnaire to assess preoperative PROs (e.g. SF-36 and EQ-5D-5 l) and patient-reported health information to screen for modifiable risk factors for perioperative complications (e.g. smoking, anxiety and depression, malnutrition, functional capacity).

This information is used during the preoperative assessment for deliberation about the patient needs and preferences and to discuss whether preoperative optimization (prehabilitation) might be of value to target certain modifiable risk factors to reduce the perioperative risk.

Postoperative we assess electronically PROMs and PREMs up to 1 year to collect relevant outcome data on patient and department levels. This information is currently structured in a dashboard for Value-Based Health Care (VBHC).

### How to use patient-reported experience measures in the context of quality improvement initiatives: the robotic experience

Robotic surgery represents the latest evolution of minimally invasive techniques, allowing to perform also complex cases in a safer manner, thanks to its technological features. During the last 20 years, the use of robotic surgery has progressively spread worldwide, covering a wide range of operations, also in the field of thoracic surgery [21]. Compared to other surgical approaches, the cost-effectiveness of robotic surgery is controversial, with consequent delays in its routine use in many realities. In Tuscany (Italy), a regional robotic program has been adopted, with 10 robotic platforms located in 5 public hospitals. The high number of robotic surgical systems available and the high volume of robotic procedures performed request the ongoing evaluation of the economic sustainability of this technology. Therefore, the systematic collection of PREMs and PROMs in robotic oncological surgery was introduced in 2018 [22].

The Tuscan experience with patient-reported experience started in 2004, based on the collaboration with the Management and Health (MeS) Lab of the Scuola Superiore Sant'Anna of Pisa. The methodology used by the MeS Laboratory for administering the survey (see Supplementary Material) to patients has been innovated over time, for adopting the last technological solutions and meeting the preferences of respondents, so moving from the postal to full-digital surveys. Currently, the patient experience is collected and returned as a continuous PREMs Observatory, still used nowadays in different healthcare systems [23].

The PREMs Observatory aims to continuously collect and real-time report data on patient experiences related to hospitalization, to contribute to the improvement of the healthcare system. Thus, the patients are enrolled on a web platform, and they receive a web link by email and/or SMS within 48 h of hospital discharge.

The anonymous results are reported via an online web-based platform and the hospital staff can log into it for the elaboration of data from the clinic, managerial and economic points of view. The nonstop collection and return of data optimize the use of the information, contextualizing it in the ongoing situation. The constant information processing system is useful to timely identify problems or weaknesses in the system, to apply changes in the process and to verify in real time the results related to these modifications.

**Figure 1:**
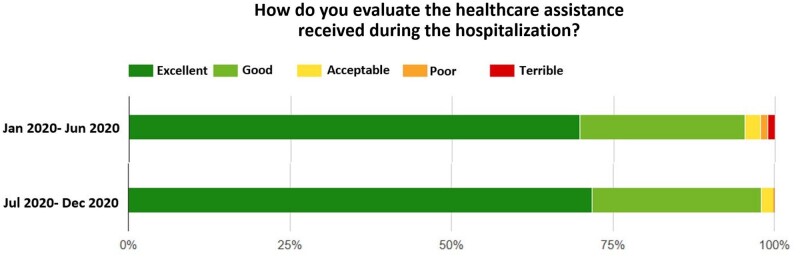
Questionnaire.

The PREM questionnaire, in use in the University Hospital of Pisa, developed by the MeS Lab, consists of 39 questions, evaluating the patient experience during the hospitalization (Fig. 1). The survey investigates from the hospital admission to the discharge, and it is divided into four sections: socio-demographic characteristics of the patient, admission, hospital stay and discharge. The survey is composed of closed-ended and open-ended questions. The closed-ended questions explore clinical details (age, sex, educational level), quietness and cleanliness of the ward, pain and emotional support, management of medical choices and clarity of information [24]. The evaluation of closed-ended questions provides healthcare benchmarking, comparing data of different units, hospitals or regions. Furthermore, the open-ended questions integrate the evaluation with additional information about the patient experience, such as observations on empathy proved by the operators and their willingness to listen or comment on the hospital organization. This section of the survey is an instrument to improve the quality of the service. The possibility to analyse patient satisfaction by PREMs represented a valuable instrument during the coronavirus disease pandemic, when substantial variations in hospital organization were required. As shown by the annual PREMs report, the constant analysis of patient experience has enabled the adaptation of hospital organization according to new pandemic-related regulations, with a focus on patient satisfaction, despite the introduction of several daily limitations. In detail, the report showed unsatisfactory healthcare assistance experienced by some patients during the hospitalization in the first part of 2020. Furthermore, the analysis of the questionnaire evidenced a negative perception regarding the communication gap and the support in facing anxieties and fears from the healthcare providers. Consequently, increased attention was paid to improving moral support and communication with the patients and an improvement in the patient perception of the quality of care was recorded during the second half of 2020.

**Figure 2:**
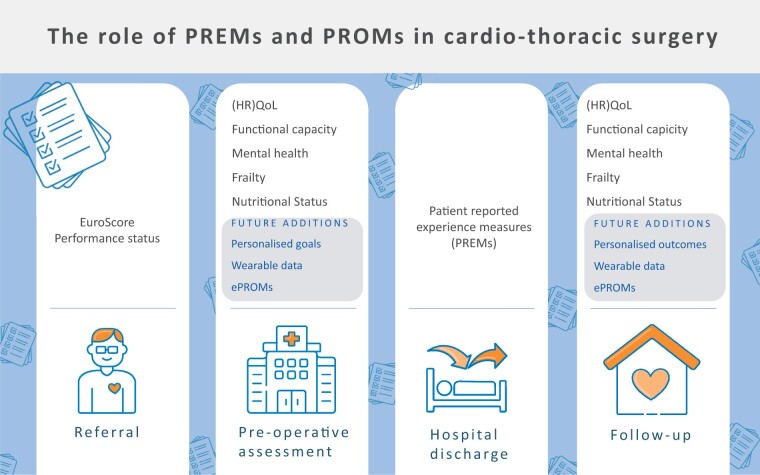
Patient perception of healthcare assistance during the coronavirus disease 2019 pandemic.

The enrolment of patients who underwent robotic surgery for PREMs survey was introduced at the University Hospital of Pisa in 2018, becoming routine since 2019. From 2019 to 2022, the overall number of patients enrolled was 2408 (92,4%), out of 2606 recruitable patients, with a rate of adhesion from the patients of 42%. Adherence to the survey from the patients has progressively increased in the last years, from 35% to 47% and it is achieved by the strong motivation characterizing the healthcare operators, due to the opportunity to obtain feedback for the work provided. Moreover, PREMs require a minimal commitment for the healthcare professionals, which are exclusively engaged in the first phase of the information and enrolment of the patients, without impacting the daily workload. Furthermore, in the last year, participation in the PREMs program has been introduced in the accreditation process of the hospital unit, with the objective of encouraging patient enrolment. The digital method of data collection, adopted in Tuscany, provides for an active and direct contribution from the patients to take the survey, by using electronic devices, also offering a realistic evaluation of the experience in hospitalization, avoiding possible influence from healthcare operators.

In Pisa, the introduction of patient-reported indicators and their regular and continuous use in clinical practice has played a crucial role in standardizing a high level of care with the optimization of the outcomes of robotic surgery, also supporting economic sustainability. A higher degree of acceptance of PREMs is achievable according to our experience, although the adoption of further strategies to increase the involvement of professionals and patient contribution should be implemented.

### Can we standardize patient-reported measures in CT surgery?

It is clear how PROMs and PREMs can effectively shape and integrate a patient-centred care (Fig. 2), but barriers and facilitators should be identified and elaborated before considering a future implementation in clinical practice. Major problems have been found in PROMs/PREMs study design, implementation, reporting and interpretation in many disciplines, as surgery is relatively naive to these outcomes.

Implementation scientists have highlighted the importance of stakeholders’ involvement and training as one of the key points to improve a successful implementation into clinical practice [25]. The incorporation of electronic completion of these measures (ePROMs) will certainly follow a clear shift towards electronic data capture driven by regulatory and practical considerations. Patient involvement in their development has demonstrated the value of the patient advocacy in implementing it in clinical practice [26]. Electronic capture of patient’s views in clinic allows real-time monitoring of symptoms, flexible scheduling of hospital appointments in response to PROM data, early detection of problems and prompt clinical intervention saving hospital visit and A&E admissions [27].

More evidence and implementation strategies are needed to determine the optimal use of ePROMs and ePREMs in specific settings such as cardio-thoracic surgery and patient populations especially when considering health literacy and privacy legislations.

## Supplementary Material

ivae025_Supplementary_Data

## Data Availability

All relevant data are within the manuscript and available on reasonable request.
