# Supplementary material for: The role of patient-reported outcome and experience measures in cardio-thoracic surgery
Source: Interdiscip Cardiovasc Thorac Surg. 2024 Mar 15;38(3):ivae025. doi: 10.1093/icvts/ivae025 (PMC11032704; doi:10.1093/icvts/ivae025)
Supplement: ivae025_Supplementary_Data [file ivae025_supplementary_data.pdf]

Appendix – Questionnaire
The questionnaire has been piloted and used in Italian only.
It is exclusively web-based: all the filters are automatic.

---

1. Who is filling in this questionnaire? [single response]
   - ☐ The patient
   - ☐ A parent/custodial parent/legal guardian
   - ☐ Another person on behalf of the patient

ACCESS TO THE HOSPITAL

2. What is the main reason why you chose this hospital? [single response]
   - ☐ I consider it the best hospital for my health problem
   - ☐ My general practitioner suggested it to me
   - ☐ The doctor I have chosen for treating my health problem is working there
   - ☐ A specialist suggested it to me
   - ☐ My relatives or friends suggested it to me
   - ☐ It is the closest one to where I live
   - ☐ I did not choose it, because I had an urgent attendance at the Emergency Department
   - ☐ Other (specify)

3. Is your general practitioner informed about your hospitalization? [single response]
   - ☐ Yes
   - ☐ No
   - ☐ I don’t know

4. [If 3 equal to yes] Did your general practitioner visit you during your hospital stay? [single response]
   - ☐ Yes
   - ☐ No, but the general practitioner spoke to me on the phone
   - ☐ No, he/she did not visit and did not contact me

5. [If 2 not equal to “I did not choose it, because I had an urgent attendance at the Emergency Department”] At the time of admission to the ward, were you welcomed with kindness and courtesy by the ward staff? [Likert scale]
   - ☐ Very much
   - ☐ Much
   - ☐ Enough
   - ☐ A little bit
   - ☐ Not at all
   - ☐ I do not know

6. Do you have any positive or negative comments on the admission to the ward? If so, use this space. [open-ended response]

HOSPITALISATION EXPERIENCE

7. During this hospital stay, do you think that the ward staff supported you in facing your fears and anxieties? [Likert scale; question n.7 includes an additional option only for the physiotherapists' choice: "I was not assisted by physiotherapists", which filters other questions (marked with a star in this form).]

	I did not have any fears or anxieties	Always	Often	Sometimes	Rarely	Never	I was not assisted by physiotherapists
doctors	☐	☐	☐	☐	☐	☐	
nurses	☐	☐	☐	☐	☐	☐	
physiotherapists	☐	☐	☐	☐	☐	☐	☐

8. During this hospital stay, do you think that the ward staff did everything possible to help you manage your pain? [Likert scale]*

	I did not have any pain	Always	Often	Sometimes	Rarely	Never
doctors	☐	☐	☐	☐	☐	☐
nurses	☐	☐	☐	☐	☐	☐
physiotherapists	☐	☐	☐	☐	☐	☐

9. During this hospital stay, did professionals, nurses and the other healthcare workers talk in front of you as if you weren't there? [Likert scale] *

	Never	Rarely	Sometimes	Often	Always
doctors	☐	☐	☐	☐	☐
nurses	☐	☐	☐	☐	☐
physiotherapists	☐	☐	☐	☐	☐

10. Do you have any positive or negative comments on the care you received by the ward staff? If so, use this space. [open-ended response]

11. During this hospital stay, were you involved by the health professionals as much as you would like in the choices related to your care? [Likert scale]
    - ☐ Always
    - ☐ Often
    - ☐ Sometimes
    - ☐ Rarely
    - ☐ Never

12. During this hospital stay, were the answers given by the staff to your questions clear? [Likert scale] *

	I did not ask any questions	Always	Often	Sometimes	Rarely	Never
doctors	☐	☐	☐	☐	☐	☐
nurses	☐	☐	☐	☐	☐	☐
physiotherapists	☐	☐	☐	☐	☐	☐

13. During this hospital stay, was easy for your family (or someone else close to you) to be informed about your health conditions? [Likert scale]
    - ☐ Always
    - ☐ Often
    - ☐ Sometimes
    - ☐ Rarely
    - ☐ Never
    - ☐ It was not necessary
    - ☐ I was alone
    - ☐ I don’t know

14. How would you rate the ability of the ward medical and nursing staff to work together? [Likert scale]
    - ☐ Very good
    - ☐ Good
    - ☐ Sufficient
    - ☐ Poor
    - ☐ Very poor
    - ☐ I do not know

15. Was the ward (room, bathroom, corridors, other common spaces, etc.) quiet? [Likert scale]
    - ☐ Very much
    - ☐ Much
    - ☐ Enough
    - ☐ A little bit
    - ☐ Not at all

16. Was the ward (room, bathroom, corridors, other common spaces, etc.) clean? [Likert scale]
    - ☐ Very much
    - ☐ Much
    - ☐ Enough
    - ☐ A little bit
    - ☐ Not at all

17. Do you have any positive or negative comments on the comfort of the ward where you stayed? If so, use this space. [open-ended response]

18. Considering the time you/your family could need to organize your return home, how much time passed between the communication of the discharge and the discharge itself? [single response]
    - ☐ More than 24 hours
    - ☐ Between 24 and 13 hours
    - ☐ Less than 12 hours

19. Do you have any positive or negative comments on your hospital experience as a whole? If so, use this space. [open-ended response]

20. Help us give value to the people who took care of you: would you like to indicate any people who impressed you with the way they treated you? If so, use this space. [open-ended response]

DISCHARGE

21. Before you left the hospital, did you receive clear information on…

	Completely clear	Clear enough	Not clear	This kind of information was not necessary for me	I did not receive any information
What to control once back home (i.e. physical activity, food, smoking,…)	☐	☐	☐	☐	☐
What drugs to take once back home	☐	☐	☐	☐	☐

22. Before you left the hospital, did you get information in writing on the drugs to take once back home (i.e. duration of the therapy, frequency of administration, …)? [single response]
    - ☐ Yes, I received information in writing
    - ☐ No, I didn't receive information in writing, only verbally
    - ☐ No, I received no information either in writing, or verbally, but they prescribed me drugs
    - ☐ No drugs were prescribed to me

23. Was the discharge letter clear? [Likert scale]
    - ☐ Very much
    - ☐ Much
    - ☐ Enough
    - ☐ A little bit
    - ☐ Not at all
    - ☐ I did not receive the discharge letter

24. Overall, how would you rate the care that you received in the ward? [Likert scale]
    a. Very good
    b. Good
    c. Sufficient
    d. Poor
    e. Very poor

25. How likely is it you would recommend this ward to relatives/friends/acquaintances having the same health problem as you? [Likert scale]
    - Yes, completely
    - Maybe
    - Not at all

ONCE AT HOME

26. Once back home, did you need home care provided by a nurse or another healthcare worker? [Multiple response]
    - Yes, by someone from the Local Health Authority (LHS)
    - Yes, I paid for someone myself
    - Yes, I needed home care but I did not receive any, neither from the LHS nor paid by me
    - No, I did not need any home care
    - No, I was moved to another hospital
    - Other [open-ended]

ABOUT YOU

27. In general, how do you consider your current health status?
    - Excellent
    - Very good
    - Good
    - Very poor
    - Poor

28. Do you have any chronic health conditions? [i.e. heart disease, stroke, cancer, diabetes, chronic respiratory disease, mental illness, muscular-skeletal disorders, digestive system disease, visual or hearing impairment, genetic disease]
    - No
    - Yes
    - I don’t know/remember

29. [if 28 equal to “yes”] Was this hospitalization related to a chronic disease that you have?
    - No
    - Yes
    - I don’t know/remember

30. Sex of the patient [single response]
    - ☐ Female
    - ☐ Male

31. [if 1 not equal to “the patient”] Sex of the respondent [single response]
    - ☐ Female
    - ☐ Male

32. Birth year of the patient [open-ended response; only 4 numbers starting with 19]

33. [if 1 not equal to “the patient”] Birth year of the respondent [open-ended response; only 4 numbers starting with 19]

34. Highest grade or level of education of the patient [single response]
    - ☐ No formal education/Primary school diploma
    - ☐ Middle school diploma
    - ☐ High school diploma
    - ☐ Academic or professional higher education
    - ☐ Master and/or doctoral studies

35. [if 1 not equal to “the patient”] Highest grade or level of education of the respondent [single response]
    - ☐ No formal education/Primary school diploma
    - ☐ Middle school diploma
    - ☐ High school diploma
    - ☐ Academic or professional higher education
    - ☐ Master and/or doctoral studies

36. Citizenship of the patient [single response; the option can be chosen in the list of all countries]

37. [if 1 not equal to “the patient”] Citizenship of the respondent [single response; the option can be chosen in the list of all the countries]
